# C-reactive protein as a possible marker for severity and mortality of COVID-19 infection 

**Published:** 2021

**Authors:** Noorollah Tahery, Mahmood Khodadost, Somayeh Jahani Sherafat, Mostafa Rezaei Tavirani, Nayebali Ahmadi, Fatemeh Montazer, Majid Rezaei Tavirani, Nosratollah Naderi

**Affiliations:** 1 *Abadan Faculty of Medical Sciences. Abadan. Iran*; 2 *School of Traditional Medicine Shahid, Beheshti University of Medical Sciences, Tehran, Iran*; 3 *Laser Application in Medical Sciences Research Center, Shahid Beheshti University of Medical Sciences, Tehran, Iran*; 4 *Proteomics Research Center, Faculty of Paramedical Sciences, Shahid Beheshti University of Medical Sciences, Tehran, Iran*; 5 *Firoozabadi Clinical Research Development Unit, Iran University of Medical Sciences, Tehran, Iran*; 6 *Faculty of Medicine, Iran University of Medical Sciences, Tehran, Iran*; 7 *Gastroenterology and Liver Diseases Research Center, Research Institute for Gastroenterology and Liver Diseases, Shahid Beheshti University of Medical Sciences, Tehran, Iran*

**Keywords:** C-reactive protein, COVID-19, Bioinformatics, Severity, Network analysis

## Abstract

**Aim:**

The present study aimed to introduce a possible biomarker to differentiate between severe and fatal conditions of COVID-19.

**Background:**

The COVID-19 pandemic, appearing as a complicated health problem, has changed the lifestyle of people in recent years. Clinical findings indicate mild, severe, and fatal conditions of this disease. Prediction of disease severity is a significant point in managing COVID-19 infection

**Methods:**

In this study, 195 differentially expressed genes (DEGs) that discriminate between fatal and severe conditions in patients were extracted from the literature and screened to determine the significant ones. The significant DEGs plus the 90 first neighbors added from the STRING database were included in the interactome using Cytoscape software v 3.7.2. The central nodes of the analyzed network were identified and assessed.

**Results:**

Ten significant DEGs were candidates for assessment, of which 9 were recognized by the STRING database. IL6, ALB, TNF, CRP, INS, MPO, C3, CXCL8, TTR, and TLR4 were determined as central nodes; IL6, CRP, and TTR were highlighted as the critical genes related to the severity of COVID-19 infection.

**Conclusion:**

CRP was identified as the best possible biomarker with levels related to the severity and fatality of COVID-19 infection.

## Introduction

 “Severe acute respiratory syndrome coronavirus-2 (SARS-CoV-2)” has arisen in recent years, triggering a significantly spreadable disease known as coronavirus disease 2019 (COVID-19) (1). This infection has remarkably altered the daily lives of people and affected various aspects of human lifestyle (2). Much documentation has presented various protocols for the treatment, diagnosis, and prevention of COVID-19 infection (3-5). 

Molecular mechanism discovery is a significant activity that can lead to improved clinical aspects of diseases. In many cases, the roles of biomolecules in the control or development of diseases are investigated (6, 7). High throughput methods are applied to assess the molecular mechanism of a wide range of disorders (8). Proteomics and genomics are two powerful, high throughput methods used in the evaluation of diseases. The identification of differentially expressed genes (DEGs) and proteins in patients can lead to the discovery of biomarkers and the determination of drug targets (9, 10). Bioinformatics is another powerful method that is tied to proteomics and genomics for explaining the events discovered by these two methods. This integration facilitates access to new biomarkers (11). 

Network analysis is a useful method for the organization and management of proteomic and genomic findings using bioinformatics tools (12). The molecular mechanisms of many diseases are investigated with network analysis. In such research, large numbers of genes or proteins are included in a connected unit as a network. In ‘scale-free’ networks, each protein or gene (that is known as a node) interacts with its neighbors in a different pattern than the other. Based on the role of the node in interactions with other nodes, the few network elements known as central nodes are differentiated from the other nodes. It is proposed that central nodes play a critical role in disease progression. They can be considered as drug targets (13-15). Hub nodes (nodes that make a large number of connections with the first neighbor nodes) are recognized as central nodes. Several diseases can be investigated using the identified hub nodes (16). There are published studies about the application of network analysis in exploring various aspects of COVID-19 infection (17, 18). In the present study, data on the fatal and severe conditions of COVID-19 patients was extracted from the literature and screened to find the DEGs to investigate with network analysis with the aim of identifying a suitable biomarker to discriminate fatal from severe COVID-19 cases. 

## Methods

Original data was extracted from the published results of Ting Shu et al. (19), who reported DEGs which discriminate fatal and severe cases of COVID-19 patients from controls. In the present study, DEGs expressed in patients with a fatal COVID-19 infection versus those with a severe case were determined among the original data. A difference of at least 1.5 FC between fatal and severe cases was considered to identify the DEGs that discriminate between the two conditions.

The final confirmed DEGs were assessed by the STRING database, and the recognized individuals were interacted by Cytoscape software. To rank the analyzed DEGs, 90 first neighbors (the least number of neighbors required to connect all queried genes) were added to the DEGs, and the interactome was created. The network was analyzed by the “NetworkAnalyzer” application of Cytoscape to determine centrality parameters for the studied nodes of the network. Four centrality parameters, degree, betweenness centrality (BC), closeness centrality (CC), and stress, were determined. The top 10% of nodes of the network based on degree value were selected as hub nodes. The top genes based on centrality and expression value were analyzed among the queried and the added first neighbors. 

## Results

**Table 1:** Differential genes that discriminate COVID-19 fatal patients from the individuals with sever condition. Sever FC and fatal FC refer to fold change in the sever and fatal condition of COVID-19 infection respectively

R	Gene	Description	Sever FC	Fatal FC	Sever FC/Fatal FC
1	CRP	C-reactive protein	6.9	15.4	2.23
2	S100A8	Protein S100-A8	3.6	8.4	2.33
3	S100A9	Protein S100-A9 1	1.1	2.6	2.36
4	IGHV1-2	Immunoglobulin heavy variable 1-2	1.4	2.2	1.57
5	GANAB	Neutral alpha-glucosidase AB	1.2	2.0	1.66
6	PSMA3	Proteasome subunit alpha type-3	1.0	1.5	1.50
7	RAB7A	Ras-related protein Rab-7a	1.1	0.7	0.64
8	TTR	Transthyretin	0.7	0.4	0.57
9	PI16	Peptidase inhibitor 16	0.6	0.4	0.67
10	CETP	Cholesteryl ester transfer protein	0.6	0.3	0.50

Ten genes among the reported 195 genes (which were represented in the original article) were determined as DEGs that discriminate between fatal and severe cases of COVID-19 (Table 1). 

The difference in gene expression value of at least 

**Figure 1:**
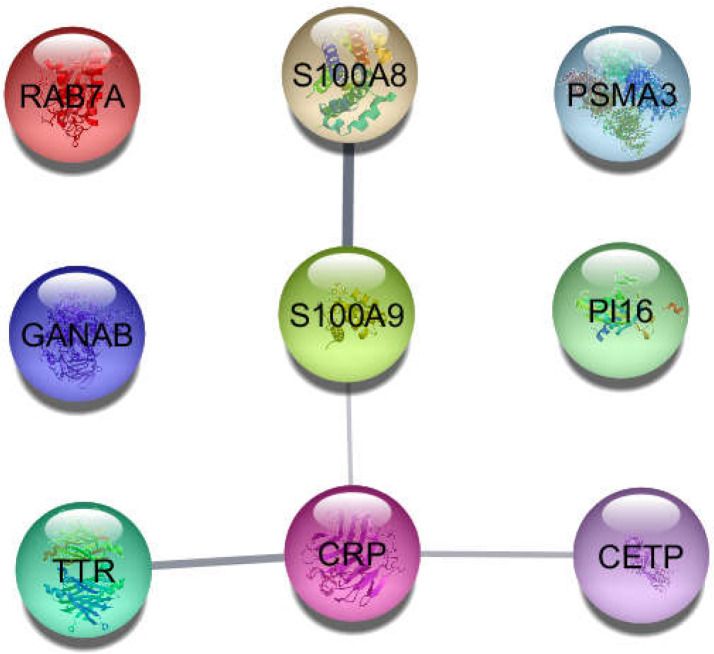
Nine recognized queried DEGs by STRING database

**Figure 2:**
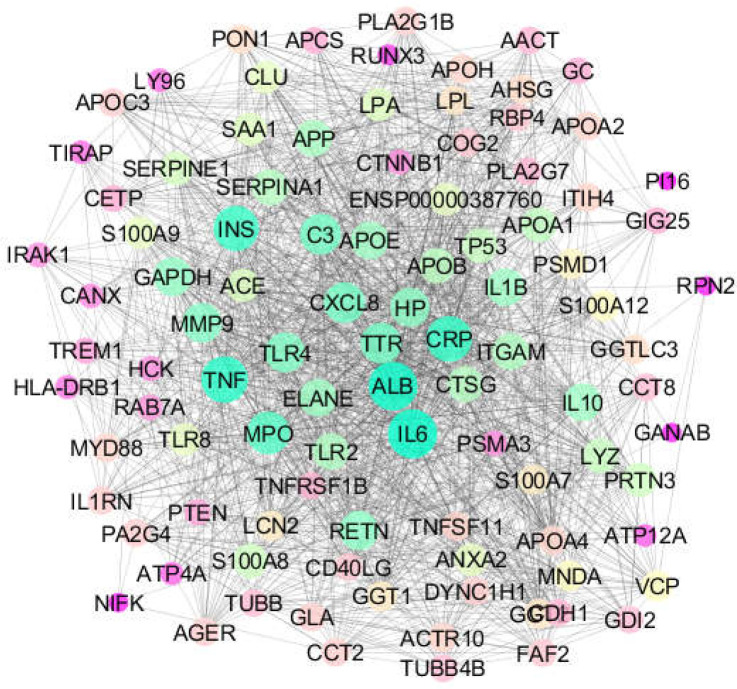
The network including 9 queried DEGs plus 90 added first neighbors which are connected by 1596 edges. The nodes are layout based on degree value color and size

1.5 FC was considered. 

As shown in Figure 1, 9 genes among the 10 queried individuals were recognized by STRING database; IGHV1-2 was the only queried DEG not recognized by STRING. Because at least 90 first neighbors were required to connect all recognized queried genes, the network was constructed with the 9 recognized queried genes plus 90 added first neighbors. The created interactome including 99 nodes and 1596 edges is represented in Figure 2. The central nodes (hubs) of the analyzed network are identified and tabulated in Table 2.

**Table 2:** Hub nodes of the assessed network. BC and CC refer to betweenness centrality and closeness centrality respectively. The queried DEGs are bolded

Gene	Degree	BC	CC	Stress
IL6	74	0.046	0.803	5010
ALB	73	0.042	0.797	4454
TNF	68	0.036	0.766	4408
CRP	66	0.029	0.748	3150
INS	65	0.033	0.737	3332
MPO	60	0.029	0.715	2968
C3	55	0.027	0.695	2716
CXCL8	55	0.017	0.690	2314
TTR	55	0.031	0.695	2484
TLR4	53	0.014	0.681	2152

## Discussion

Data analysis revealed that 10 of the 195 studied genes can be considered as discriminative factors to differentiate fatal cases from severe cases of COVID-19 infection. As shown in Table 1, 6 genes, namely CRP, S100A8, S100A9, IGHV1-2, GANAB, and PSMA3, are up-regulated and 4 (RAB7A, TTR, PI16, and CETP) are down-regulated. Among these 10 dysregulate genes, two DEGs (CRP and TTR) appeared as central nodes in the network analysis, and IL6 as an added first neighbor was identified as the top hub node. It can be concluded that CRP, TTR, and IL6 are the critical genes that are able to discriminate fatal from severe cases of COVID-19 infection in the patients. 

As reported by researchers, CRP levels are positively correlated with the severity of COVID-19 and lung lesions. It has also been reported that the plasma level of CRP is not a biomarker to predict infection severity. Another investigation reported that a 10-20 µg/ml elevation in CRP can be considered a diagnostic factor for a mild case of COVID-19 infection (20-22). Ethem Acar et al. showed that inflammatory parameters such as lymphocyte-to-C-reactive protein ratio (LCRP), systemic immune inflammation index (SII), and neutrophil-to-lymphocyte ratio (NLR) can be considered as parameters associated with COVID-19 disease severity (23). 

Transthyretin levels in COVID-19 patients were searched in the literature, but no valid documents were found. As shown in Table 1, this protein was downregulated in the plasma of patients with both severe and fatal infections. The findings indicated that severity of disease is negatively associated with TTR levels; however, it seems that checking TTR levels is a useful tool for follow-up of disorder progression.

EA Comes and H Haghbayan published a systematic review and meta-analysis regarding the elevation of IL6 levels in correlation with COVID-19 infection severity, and ZS Ulhaq and JV Soraya introduced IL6 as a potential biomarker for progression of COVID-19 disease (24). H Zhang et al. pointed to IL6 as an early identification indicator of severe COVID-19 condition (25). Inflammatory biomarkers in COVID-19 infection have been highlighted in many investigations. It is emphasized that these biomarkers are related to severity, mortality, neurocognitive impairment, psychopathology, and other aspects of the disease (26-28).

Each of the three introduced possible biomarkers, i.e. CRP, TTR, and IL6, is characterized with its specific property. IL6 appeared as a central node among the added first neighbors, and elevated levels of it are related to infection severity. In contrast, TTR was a queried DEG, and its failed levels are attributed to the progression of COVID-19 disease. CRP was a queried DEG and a highly expressed gene found to be associated with COVID-19 severity. CRP also appeared as a central node in the created network. It can be concluded that CRP level calibration may be a useful tool for predicting and reflecting the severity and mortality rates of COVID-19 infection.

## Conclusion

The findings indicate that three critical proteins (CRP, IL6, and TTR) are related to the severity and mortality rate of COVID-19 infection. It seems that CRP is the best candidate to predict disease development. The results also indicated a positive correlation between CRP and COVID-19 severity in patients. It is suggested that CRP level calibration can be a useful tool in managing COVID-19 patients.

## Conflict of interests

The authors declare that they have no conflict of interest.
